# The Regulatory Impact of CFLAR Methylation Modification on Liver Lipid Metabolism

**DOI:** 10.3390/ijms25147897

**Published:** 2024-07-19

**Authors:** Chen Ye, Wen Jiang, Ting Hu, Jichao Liang, Yong Chen

**Affiliations:** National & Local Joint Engineering Research Center of High throughput Drug Screening Technology, Hubei Province Key Laboratory of Biotechnology of Chinese Traditional Medicine, College of Health Science and Engineering, Hubei University, Wuhan 430062, China; 202311107010121@stu.hubu.edu.cn (C.Y.); 202111107010111@stu.hubu.edu.cn (W.J.); 202211107010125@stu.hubu.edu.cn (T.H.)

**Keywords:** CFLAR, lipid accumulation, methylation, NAFLD, PRMT1, pJNK1

## Abstract

Non-alcoholic fatty liver disease (NAFLD) has emerged as the leading cause of chronic liver disease worldwide. Caspase 8 and FADD-like apoptosis regulator (CFLAR) has been identified as a potent factor in mitigating non-alcoholic steatohepatitis (NASH) by inhibiting the N-terminal dimerization of apoptosis signal-regulating kinase 1 (ASK1). While arginine methyltransferase 1 (PRMT1) was previously reported to be associated with increased hepatic glucose production, its involvement in hepatic lipid metabolism remains largely unexplored. The interaction between PRMT1 and CFLAR and the methylation of CFLAR were verified by Co-IP and immunoblotting assays. Recombinant adenoviruses were generated for overexpression or knockdown of PRMT1 in hepatocytes. The role of PRMT1 in NAFLD was investigated in normal and high-fat diet-induced obese mice. In this study, we found a significant upregulation of PRMT1 and downregulation of CFLAR after 48h of fasting, while the latter significantly rebounded after 12h of refeeding. The expression of PRMT1 increased in the livers of mice fed a methionine choline-deficient (MCD) diet and in hepatocytes challenged with oleic acid (OA)/palmitic acid (PA). Overexpression of PRMT1 not only inhibited the expression of genes involved in fatty acid oxidation (FAO) and promoted the expression of genes involved in fatty acid synthesis (FAS), resulting in increased triglyceride accumulation in primary hepatocytes, but also enhanced the gluconeogenesis of primary hepatocytes. Conversely, knockdown of hepatic PRMT1 significantly alleviated MCD diet-induced hepatic lipid metabolism abnormalities and liver injury in vivo, possibly through the upregulation of CFLAR protein levels. Knockdown of PRMT1 suppressed the expression of genes related to FAS and enhanced the expression of genes involved in FAO, causing decreased triglyceride accumulation in OA/PA-treated primary hepatocytes in vitro. Although short-term overexpression of PRMT1 had no significant effect on hepatic triglyceride levels under physiological conditions, it resulted in increased serum triglyceride and fasting blood glucose levels in normal C57BL/6J mice. More importantly, PRMT1 was observed to interact with and methylate CFLAR, ultimately leading to its ubiquitination-mediated protein degradation. This process subsequently triggered the activation of c-Jun N-terminal kinase 1 (JNK1) and lipid deposition in primary hepatocytes. Together, these results suggested that PRMT1-mediated methylation of CFLAR plays a critical role in hepatic lipid metabolism. Targeting PRMT1 for drug design may represent a promising strategy for the treatment of NAFLD.

## 1. Introduction

Nonalcoholic fatty liver disease (NAFLD) refers to a prevalent metabolic syndrome characterized by hepatic abnormalities and the accumulation of excessive triglycerides in hepatocytes, representing the early stage and progressive inducement from steatosis to steatohepatitis [1,2,3]. Meanwhile, inflammatory cytokines promote the activation of liver resident Kupffer cells (KCs) and recruit circulating mononuclear-derived macrophages to aggregate in the liver. The activation of innate immunity further promotes the infiltration and accumulation of inflammatory cells in the liver, exacerbating inflammation and causing damage to the liver. The activation of hepatic stellate cells (HSCs) is the foundation for ultimately leading to liver fibrosis. Both environmental and genetic factors contribute to NAFLD development, while the genetic determinants predisposing individuals to hepatic steatosis are poorly defined [4,5,6]. Caspase 8 and fadd-like apoptosis regulator (CFLAR) was previously identified as a key genetic inhibitor of hepatitis deterioration [7,8]. Delivery of CFLAR and its S1 fragment in mice and non-human primates alleviated hepatic lipid deposition and liver injury. However, the post-translational modifications of CFLAR and their consequent impact on CFLAR’s biological function remain largely unexplored. 

Epigenetic modifications have been recognized as pivotal in the pathogenesis of various metabolic liver diseases [9,10,11]. Among these functional proteins carrying such a process, protein arginine methylation catalyzed by the arginine methyltransferase (PRMT) family is a ubiquitous post-translational modification [12,13]. PRMT1 (also known as Hrmt1/2, Mrmt1) contributes to most protein arginine methylation, including arginine mono-methylarginine (MMA) and asymmetric dimethylarginine (ADMA). Moreover, PRMT1 shares a highly conserved protein sequence among species, revealing its evolutionary conservatism and essential function. The “VATLANGMSL” sequences located in exon 2 are rich in hydrophobic groups and serve as a nuclear transfer signal (NES) sequence [14,15]. Therefore, transcripts differ in the final cell sub-localization.

PRMT1 was initially identified as a critical oncogene in various types of human cancers [16,17,18,19]. Recently, PRMT1 was revealed to play a pivotal role in metabolic diseases, including insulin resistance and obesity. Methylation of FoxO1 (Forkhead box protein O1) by PRMT1 regulated its phosphorylation and impacted blood glucose levels in mice [20]. Silencing of PRMT1 has been shown to improve PA-induced lipid accumulation in hepatocytes [21]. As a key mediator molecule of glycolipid metabolism, PGC-1α (peroxisome proliferator*s*-activated receptor γ coactivator alpha) was also reported to be the methylation substrate of PRMT1, thus regulating the thermogenesis in adipocytes [22,23]. Intriguingly, AAV-mediated knockdown of PRMT1 was reported to exacerbate liver lipid metabolism disorder in high-fat diet (HFD)-induced mice by modulating the lipid oxidation process [24]. 

Although PRMT1 is almost guaranteed to be involved in multiple hepatic diseases based on the numerous studies mentioned above, its precise methyl group receptor and physiological function remain disputed [25,26]. Here, we show that PRMT1 physiologically interacts with CFLAR and directly enhances the ADMA levels of CFLAR, thereby affecting the CFLAR-JNK axis and subsequently promoting lipid deposition in hepatocytes. Overexpression of PRMT1 significantly promotes lipid accumulation through the CFLAR-JNK axis in mouse primary hepatocytes, while knockdown of PRMT1 alleviates this metabolic disorder in OA/PA-treated primary hepatocytes. In vivo, knockdown of hepatic PRMT1 can markedly alleviate lipid accumulation and liver injury induced by the MCD diet in mice. Under normal physiological conditions, short-term overexpression of PRMT1 in the normal mouse liver upregulates the levels of serum TG and TC, although no obvious changes in hepatic TG content are observed. These findings suggest that protein methylation of CFLAR by PRMT1 plays a crucial role in the progression of NAFLD, and targeting CFLAR methylation may represent a promising therapeutic strategy for the treatment of NAFLD. 

## 2. Results

### 2.1. Hepatic Protein Expression of PRMT1 and CFLAR Are Regulated by Fasting/Refeeding Cycles and Are Dysregulated in Diet-Induced Mouse Models of NAFLD

**Figure 1:**
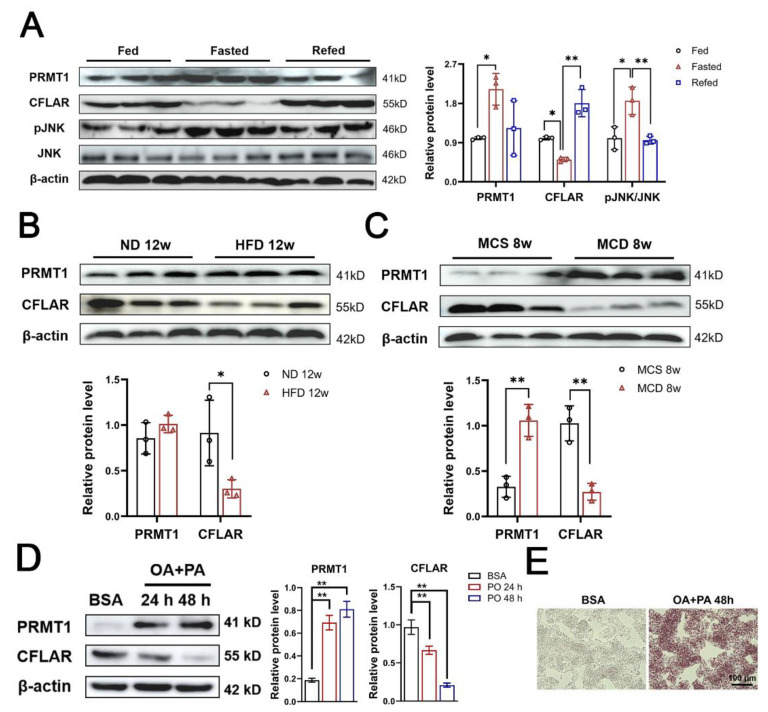
Hepatic PRMT1 expression is regulated by nutritional status and is increased in diet-induced nonalcoholic steatohepatitis mouse models. (**A**) Western blot analysis of the protein expression of PRMT1, CFLAR, p-JNK, and JNK from mice livers under ad libitum feeding, 48 h fasting, and 12 h refed conditions (*n* = 3/group). Right panel, quantification of data represented in the left panel by ImageJ. (**B**) Western blot analysis of the protein expression of PRMT1 and CFLAR in livers from mice (*n* = 3/group) fed a normal chow diet (ND) or high-fat diet (HFD) for 12 weeks. (**C**) Western blot analysis of the protein levels of PRMT1 and CFLAR in livers of mice (*n* = 3) fed a methionine–choline-sufficient (MCS) or methionine–choline-deficient (MCD) diet for 8 weeks. Bottom panels, quantification of data represented in up panel of B and C. (**D**) Western blot analysis of the protein levels of PRMT1 and CFLAR in HepG2 cells treated with BSA or 0.25 mM OA + 0.5 mM PA (PO) for 24 h and 48 h, respectively. (**E**) Oil red O staining images of HepG2 cells under the PO model for 48 h. Right panel, quantification of data represented in the left panel. All expressions are normalized to β-actin. Two-tailed Student’s test and one-way ANOVA test were used. * *p* < 0.05, ** *p* < 0.01, all data are shown as the mean ± SD.

To test whether the expression of PRMT1 and CFLAR can be regulated by different cyclic nutritional status, mice were subjected to a fasting/refeeding cycle. We found that prolonged fasting induced the protein expression of PRMT1 and phosphorylated JNK(p-JNK) in the livers of normal mice, while this effect gained a downward trend by refeeding (Figure 1A). An opposite regulatory pattern on protein expression of CFLAR was observed (Figure 1A), indicating a strong correlation between PRMT1 and CFLAR. To further investigate the expression of PRMT1 and CFLAR under pathological conditions, HFD and methionine–choline-deficient (MCD) diet-induced mouse models of NAFLD were established. The results of Western blotting indicated that the expression of PRMT1 was significantly increased in the livers of MCD diet-induced mice and showed a modest trend to be upregulated in the livers of HFD-induced mice. In contrast, the protein expression of CFLAR was dramatically decreased in the livers of mice fed HFD or the MCD diet compared with those fed a chow diet. (Figure 1B,C). In addition, exposure to PA/OA promoted the expression of PRMT1 in HepG2 but, on the contrary, suppressed the expression of CFLAR (Figure 1D). Meanwhile, oil red O staining showed the accumulation of lipids in HepG2 cells treated with 48 hours of PA/OA (Figure 1E). These data strongly suggested that the expression of PRMT1 and CFLAR were inversely regulated in response to nutritional status and cycles and that the dysregulation of these two genes in hepatocytes may contribute to MCD-diet-induced lipids accumulation in the liver. 

### 2.2. Overexpression of PRMT1 Promotes Lipid Accumulation in Primary Hepatocytes and HepG2 Cells via the CFLAR-JNK Axis

**Figure 2:**
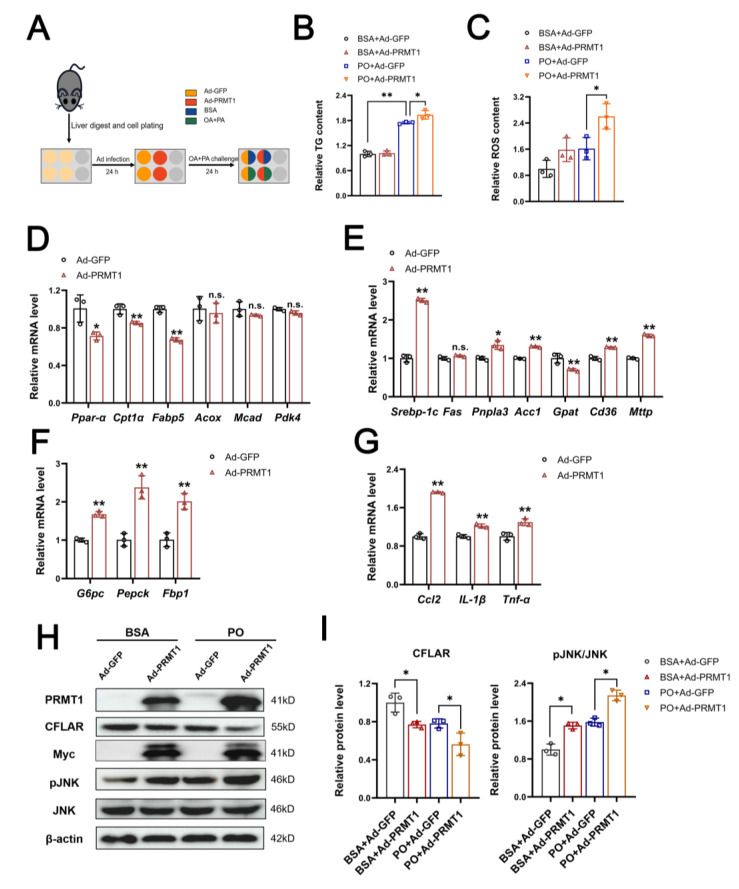
Overexpression of PRMT1 exacerbates the disorder of lipid metabolism in primary hepatocytes through the CFLAR-JNK axis. (**A**) The flowchart shows the steps of cell processing. Mouse primary hepatocytes were infected with Ad-GFP/Ad-PRMT1 for 24 h, following a 24-hour PO (0.25 mM OA + 0.5 mM PA) challenge (**B**) Analysis of triglyceride (TG) content and lipid accumulation in primary hepatocytes as treated in (**A**). (**C**). ROS levels in primary hepatocytes treated as in (**A**). (**D**–**G**) Primary hepatocytes were infected with Ad-PRMT1 or Ad-GFP in the presence of BSA, mRNA expression of genes involved in lipid oxidation (**D**), de novo synthesis and fatty acid uptake (**E**), gluconeogenesis (**F**), and inflammation (**G**) were analyzed by RT-qPCR. (**H**,**I**) Western blot analysis of PRMT1 and the CFLAR-JNK axis in primary hepatocytes in the presence/absence of PO and the corresponding protein grayscale quantitative statistics. All expressions are normalized to β-actin. A two-tailed Student’s test and a one-way ANOVA test were used. * *p* < 0.05, ** *p* < 0.01, n.s. means no significance, all data are shown as the mean ± SD.

**Figure 3:**
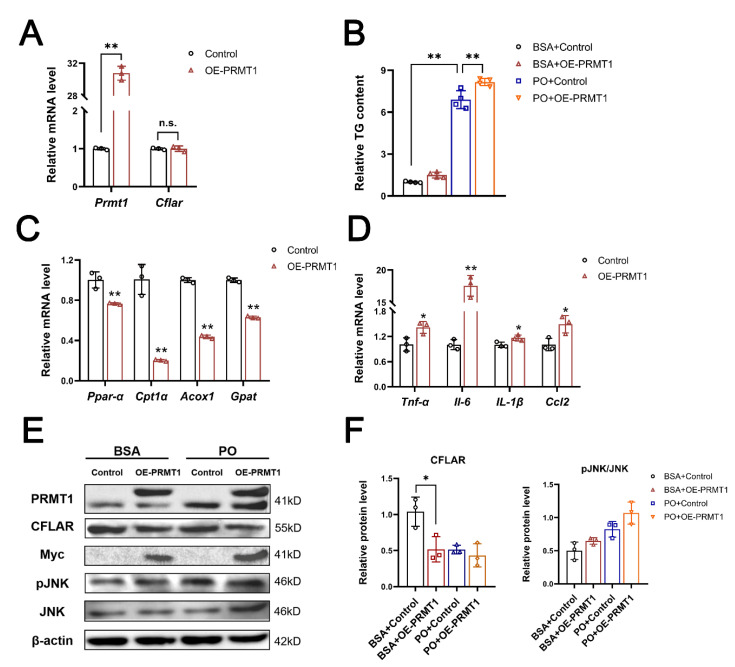
Overexpression of PRMT1 promotes lipid accumulation in HepG2 cells through the CFLAR-JNK axis. (**A**) Relative mRNA expressions of Prmt1 and Cflar in HepG2 cells transfected with empty control vector or pCMV6-PRMT1 (OE-PRMT1) for 48 h (*n* = 3). (**B**) The triglyceride (TG) content in HepG2 cells transfected with the indicated plasmid for 24 h, following another 24 h of BSA or PO treatment (*n* = 4). (**C**,**D**) Relative mRNA expressions of genes involved in fatty acid oxidation (**C**) and inflammation (D) in HepG2 cells transfected with the indicated plasmid in the presence of BSA. (**E**) HepG2 cells were treated as in (**B**), and the protein levels of PRMT1 and the CFLAR-JNK axis were analyzed by Western blot. (**F**) Quantification of protein expression represented in E. All expressions are normalized to β-actin. A two-tailed Student’s test and a one-way ANOVA test were used. * *p* < 0.05, ** *p* < 0.01, n.s. means no significance, all data are shown as the mean ± SD.

To further confirm whether PRMT1 is involved in hepatic lipid metabolism, we investigated the effect of the transfection of hepatocytes with adenovirus expressing PRMT1 on lipid metabolism. The liver of male mice was digested, and primary liver cells were plated into a six-well plate by centrifugation. Then, the cells were infected with Ad-GFP/Ad-PRMT1 for 24 hours, following another 24-hour PO challenge. (Figure 2A). As shown in Figure 2B,C, adenovirus-mediated overexpression of PRMT1 did not affect TG content or ROS levels under basal conditions but significantly increased TG content and ROS production in the primary hepatocytes treated with PA/OA (PO). Consistent with the observed phenotype, forced expression of PRMT1 inhibited the expression of genes involved in fatty acid oxidation but induced the expression of genes related to fatty acid synthesis and transport (Figure 2D,E). The ectopic expression of PRMT1 also enhanced the expression of gluconeogenic genes and proinflammatory cytokine genes, including *G6pc* (glucose-6-phosphatase), *Pepck* (phosphoenolpyruvate carboxykinase), *Fbp1* (fructose-1,6-bisphosphatase 1), *Ccl2* (chemokine ligand 2), *IL-1β* (interleukin 1 beta), and *Tnf-α* (tumor necrosis factor-α) (Figure 2F,G). Additionally, we studied the effect of PRMT1 on CFLAR/JNK pathway activation. The results revealed that adenovirus-mediated overexpression of PRMT1 significantly inhibited the protein expression of CFLAR and promoted the phosphorylation of JNK under both basal and lipid overload conditions (Figure 2H,I). Furthermore, we tested whether PRMT1 could regulate lipid accumulation in HepG2 cells. Consistent with the findings observed above, overexpression of PRMT1 significantly induced lipid deposition in PA/OA-challenged HepG2 cells, a typical humanized cell line, despite this treatment slightly but not significantly increasing triglyceride content under normal conditions (Figure 3A,B). Similar results were observed in the mRNA expression of fatty acid oxidation and proinflammatory cytokine-related genes, as well as the protein expression of CFLAR and p-JNK (Figure 3C–F). These results indicated that PRMT1 could regulate lipid metabolism in hepatocytes via the CFLAR/JNK axis.

### 2.3. Knockdown of Hepatic PRMT1 Alleviates Methionine–Choline-Deficient Diet-Induce Nonalcoholic Steatohepatitis in Mice

**Figure 4:**
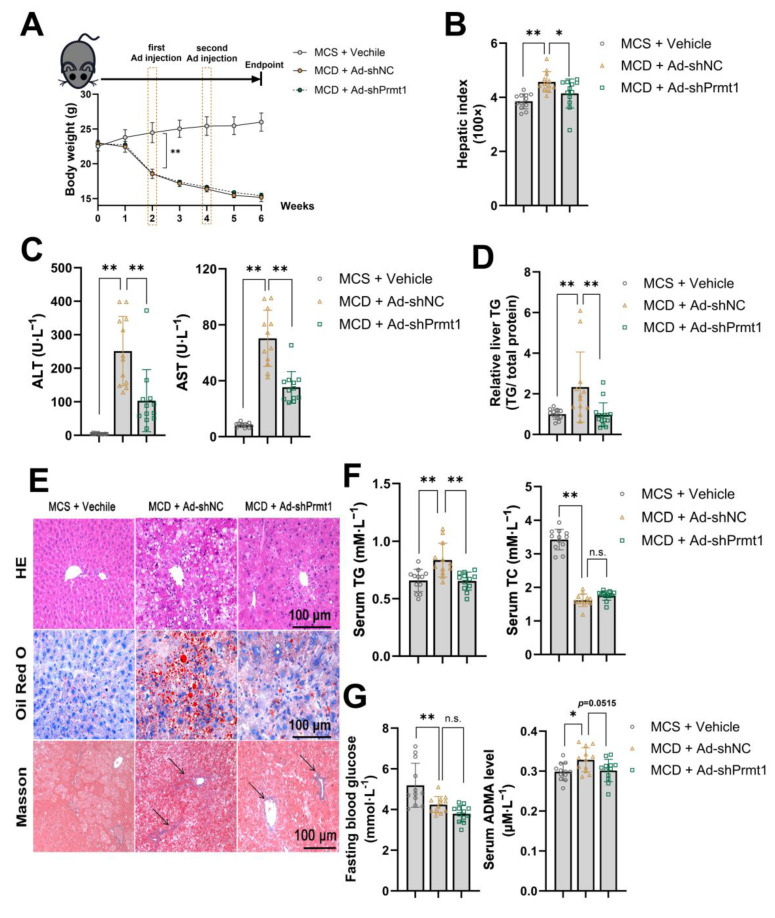
Knockdown of liver PRMT1 alleviates mice metabolism disorder induced by the methionine–choline-deficient diet. (**A**) Mice were divided into three groups as follows: MCS + Vechile, MCD + Ad-shNC, and MCD + Ad-shPrmt1. Body weight (g) of mice under six weeks of feeding. The infection of recombinant adenovirus was scheduled to start in the second and fourth weeks (*n* = 12 for each). (**B**) Hepatic index (liver weight/body weight, 100×) at the endpoint of 6 weeks of feeding (*n* = 12 for each). (**C**) AST and ALT levels in serum (*n* = 12). (**D**) Mice liver relative liver TG content (TG content/total protein), 100% of control (*n* = 12) (**E**) Images of liver staining of HE, oil red O, and masson, with black arrow pointing the bridging between repaired portal area and sinus space (scale bar 100 μm). (**F**) Serum TG and TC levels (*n* = 12). (**G**) Fasting blood glucose and serum total ADMA levels detected by the ELISA kit (*n* = 12). A two-tailed Student’s test and a one-way ANOVA test were used. * *p* < 0.05, ** *p* < 0.01, n.s. means no significance, all data are shown as the mean ± SD.

Because of the substantial differences observed in the livers of the mice fed the MCD diet, we were keen to investigate the potential therapeutic effects of interfering with PRMT1. Thus, we induced a state of hepatic lipid metabolism disorder by feeding the mice MCS or MCD diets for six weeks and administered PRMT1-interfering adenovirus (Ad-shPRMT1) via tail vein injection in the second and fourth weeks. Materials were collected at the end of the experiment after an overnight fast for analysis. The MCD diet significantly affected the body weight of mice and caused early NASH pathological manifestations. However, knockdown of hepatic PRMT1 did not affect body weight under the MCD model but significantly downregulated the liver index (liver weight/body weight) at the endpoint of the experiment (Figure 4A,B). The MCD diet induced a significant increase in serum AST/ALT, indicating liver damage, which was markedly reversed in the PRMT1 knockdown group (Figure 4C). Additionally, Ad-shPRMT1 effectively relieved the accumulation of triglycerides in the liver (Figure 4D). Ad-shPRMT1 simultaneously improved hepatic histopathological features by reducing the number of ballooning hepatocytes, oil red O staining areas, and the size of liver collagen repair areas (Figure 4E). Meanwhile, Ad-shPRMT1 treatment effectively interfered with the tendency of bridging between the repaired portal area and sinus space (black arrow pointing), which typically implies a higher NASH-associated liver fibrosis rating. These histopathological changes all demonstrated that interfering with the expression of PRMT1 under the MCD pathological state could effectively alleviate and delay the onset and progression of NASH. While the total triglycerides were effectively reduced, it failed to show a significant decrease in total cholesterol, fasting blood glucose, or total asymmetric dimethylarginine levels (*p* = 0.0515) in the serum statistically (Figure 4F,G). Relatively low levels of fasting blood glucose and serum cholesterol may attribute the success to the inconspicuous phenomenon of change caused by the MCD diet.

**Figure 5:**
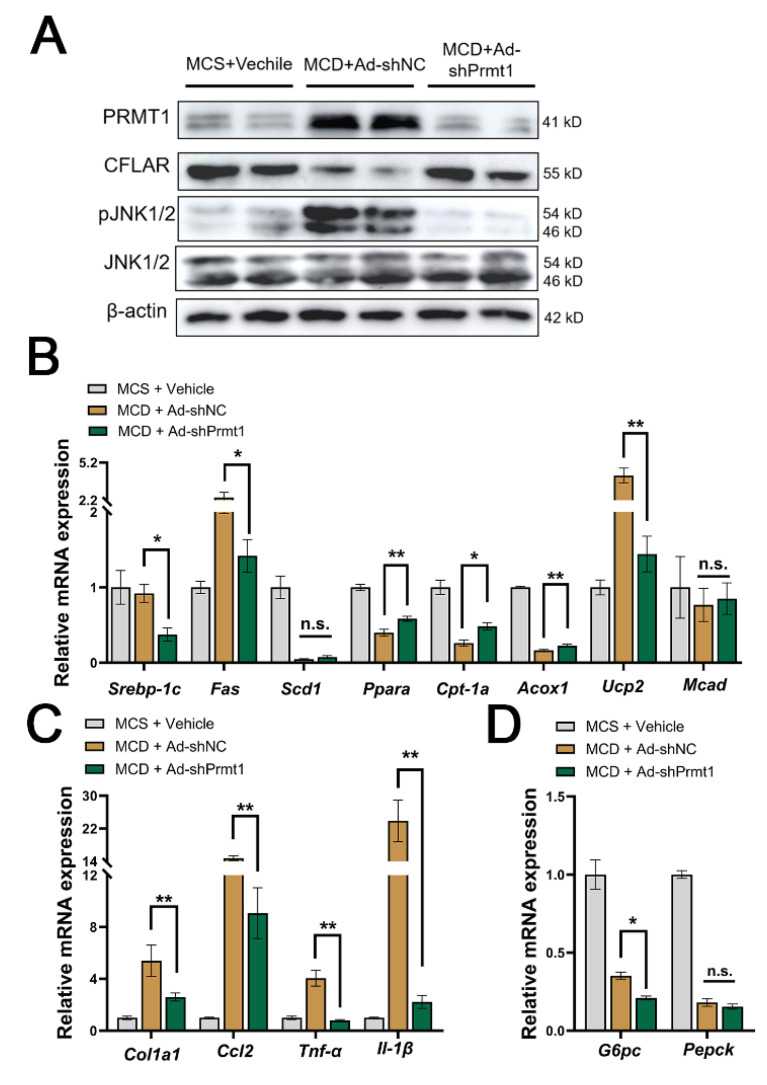
Knockdown of liver PRMT1 alleviates mice metabolism disorder induced by the methionine–choline-deficient diet via the CFLAR-JNK axis. The liver tissue was preserved in liquid nitrogen and fully lysed using RIPA or trizol reagent. (**A**) Representative images of mice liver protein expressions, including PRMT1, CFLAR, JNK, p-JNK, and actin. mRNA expression of genes involved in lipid oxidation, de novo synthesis (**B**), inflammation, cell chemotaxis, and collagen neogenesis (**C**), and gluconeogenesis (**D**) were analyzed by RT-qPCR. All expressions are normalized to β-actin. A two-tailed Student’s test and a one-way ANOVA test were used. * *p* < 0.05, ** *p* < 0.01, n.s. means no significance, all data are shown as the mean ± SD.

Because of the observations mentioned above, we extracted liver proteins and total RNA for the detection of gene expression levels. As the results show, PRMT1 and p-JNK were both strongly induced by the MCD model, accompanied by a strong inhibition of CFLAR expression. However, knockdown of PRMT1 effectively restored CFLAR protein levels and reduced JNK phosphorylation levels (Figure 5A). At the transcriptional level, Ad-shPRMT1 significantly inhibited the expression of lipid synthesis-related genes including *Srebp-1c* (Sterol regulatory element binding proteins-1c) and *Fas* (fatty acid synthase), promoted the expression of lipid oxidation-related genes including *Ppar-a* (peroxisome proliferator-activated receptor), *Cpt-1a* (arnitine palmitoyltransferase 1), and *Acox1* (acyl-coenzyme A oxidase 1), and had no significant effect on the expression of *Scd1* (Stearoyl-CoA desaturase 1) and *Mcad* (medium chain aryl-CoA dehydrogenase) (Figure 5B). Genes representing inflammation, collagen repair, and chemotactic factors under the NASH models were also significantly inhibited including *Col1a1* (collagen type I alpha 1 chain), *Ccl2*, *Tnf-α*, and *Il-1β* (Figure 5C). Genes involved in gluconeogenesis—*G6pc*—were significantly inhibited, while *Pepck* was not influenced (Figure 5D). Indeed, an MCD diet can lead to decreased fasting blood glucose and increased insulin sensitivity in mice.

### 2.4. Ad-shPRMT1 Alleviates Lipid Accumulation in Mouse Primary Hepatocytes

**Figure 6:**
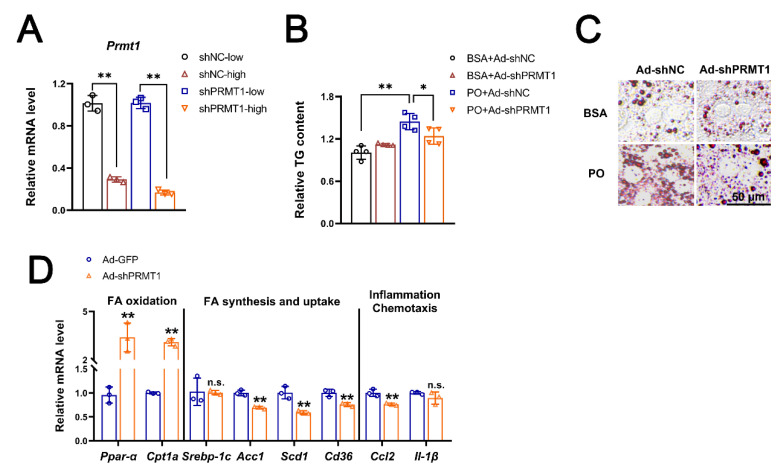
Knockdown of PRMT1 mitigates metabolic disorder in primary hepatocytes. (**A**) Relative mRNA expression of Prmt1 in primary hepatocytes infected by Ad-shNC or Ad-shPRMT1 at a low dose (1 × 10^9^ pfu) and high dose (2 × 10^9^ pfu) for 24 h. (**B**) Primary hepatocytes were infected by Ad-shNC or Ad-shPRMT1 (1 × 10^9^ pfu) for 24 h and exposed to 0.25 mM OA + 0.5 mM PA (PO) challenge for another 24 h. The intracellular triglyceride (TG) content was measured. (**C**) Oil red O staining in primary hepatocytes treated as in (**B**) (scale bar = 50 μm). (**D**) Relative mRNA expression of genes involved in fatty acid metabolism in primary hepatocytes after being infected with Ad-shNC or Ad-shPRMT1 for 48 h in the presence of PA. All expressions are normalized to β-actin. A two-tailed Student’s test and a one-way ANOVA test were used. * *p* < 0.05, ** *p* < 0.01. n.s. means no significance, All data are shown as the mean ± SD.

We further confirmed our conclusion in cells cultured in vitro. Adenovirus of different doses showed good infection and knockdown efficiency in primary liver cells extracted from normal mice (Figure 6A and Figure S1C,D). Meanwhile, Ad-shPRMT1 significantly reduced the phenomenon of lipid accumulation in primary hepatocytes under PA/OA conditions (Figure 6B,C) compared with Ad-shNC. At the transcriptional level, Ad-shPRMT1 significantly promoted the expression of genes related to fatty acid oxidation (*Ppar-a*, *Cpt-1a*), inhibited the expression of genes related to fatty acid synthesis and uptake *Acc1* (acetyl-CoA carboxylase 1), *Scd1*, and *Cd36* (cluster of differentiation 36), suppressed the expression of cell chemotaxis genes *Ccl2*, and had no significant effect on the expression of *Srebp-1c*, *Il-1β* (Figure 6D). In conclusion, interfering with PRMT1 at the level of cultured mouse primary hepatocytes can also demonstrate a positive effect in alleviating lipid accumulation.

### 2.5. PRMT1 Methylates CFLAR and Promotes Its Degradation by Poly-Ubiquitination in Primary Hepatocytes

**Figure 7:**
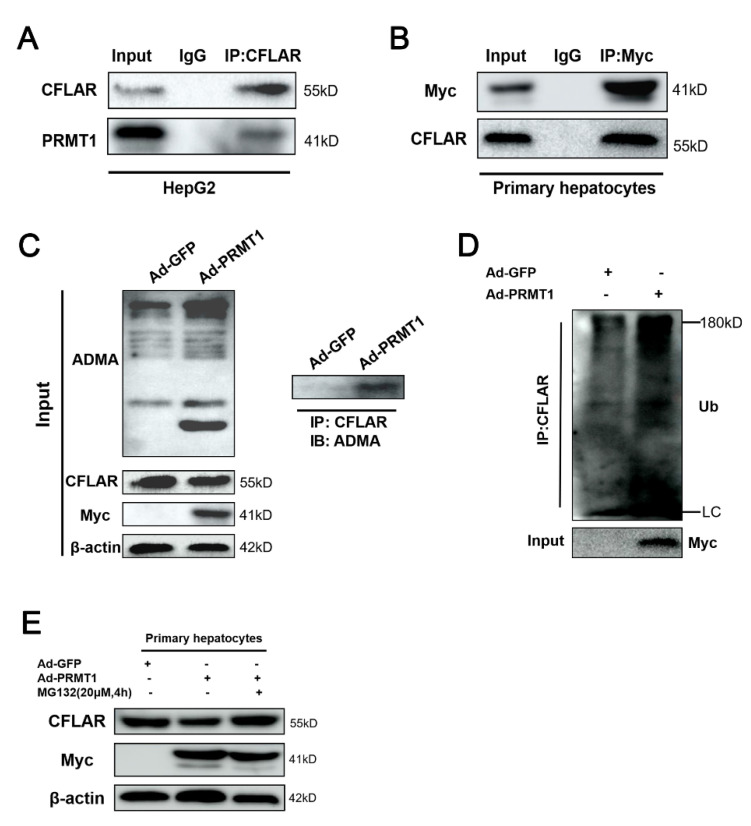
PRMT1 methylates CFLAR and regulates its degradation by poly-ubiquitination. (**A**,**B**) Representative co-immunoprecipitation (CO-IP) analysis was used to identify the endogenous interaction between PRMT1 and CFLAR in HepG2 by performing immunoprecipitation of total CFLAR (**A**). (**B**) Total lysis of primary hepatocytes infected by Ad-PRMT1 was enriched by Myc-tag antibodies and subsequently eluted, and the precipitate was detected for Myc and CFLAR, while rabbit or mouse IgG was used as the control. (**C**) IP and Western blot analysis of the ADMA levels of CFLAR in primary hepatocytes infected with Ad-GFP or Ad-PRMT1 for 48 h. (**D**) IP and Western blot analysis of the ubiquitination degree of CFLAR in primary hepatocytes treated with 20 μM MG132 for 4 h before the cell was collected. (**E**) Western blot analysis of the protein levels of CFLAR and PRMT1 in primary hepatocytes infected with Ad-GFP or Ad-PRMT1 in the presence/absence of 20 μM MG132.

Given the fact that the expression patterns of PRMT1 and CFLAR were opposite under both physiological and pathological conditions, we speculated that a direct association between PRMT1 and CFLAR might exist in hepatocytes. To verify this hypothesis, we performed co-immunoprecipitation (CO-IP) assays in HepG2 cells. The results clearly indicated a significant interaction between endogenous PRMT1 and CFLAR in HepG2 cells (Figure 7A). Consistent with the results obtained in HepG2 cells, a strong interaction between PRMT1 and endogenous CFLAR was observed in primary hepatocytes (Figure 7B). We next investigated the direct methylation of CFLAR by PRMT1. As shown in Figure 7C, adenovirus-mediated overexpression of PRMT1 markedly promoted total ADMA levels, suggesting increased ADMA-containing proteins (Figure 7C, left panel). Indeed, we found that PRMT1 overexpression dramatically increased the asymmetric dimethylarginine of CFLAR in primary hepatocytes, as evidenced by a CO-IP assay (Figure 7C, right panel). Additionally, we also found that different concentrations of ADMA treatment (0.3/0.6/1 μM, HY-113216, MCE) significantly reduced the protein levels of CFLAR (Supplementary Figure S2). So, to verify whether the PRMT1-mediated methylation of CFLAR was attributed to its ubiquitination-mediated protein degradation, we performed a ubiquitination assay and found that overexpression of PRMT1 led to a significant increase in ubiquitination of CFLAR (Figure 7D) in primary hepatocytes. Furthermore, the results from Western blotting indicated that overexpression of PRMT1 decreased the protein levels of CFLAR, but further treatment with MG132, a proteasome inhibitor, reversed this inhibitory effect (Figure 7E). These data suggest that post-translational modification of CFLAR plays a critical role in the regulation of hepatic lipid metabolism by PRMT1. 

### 2.6. Hepatic Overexpression of PRMT1 Promotes VLDL Secretion in C57BL/6J Mice

**Figure 8:**
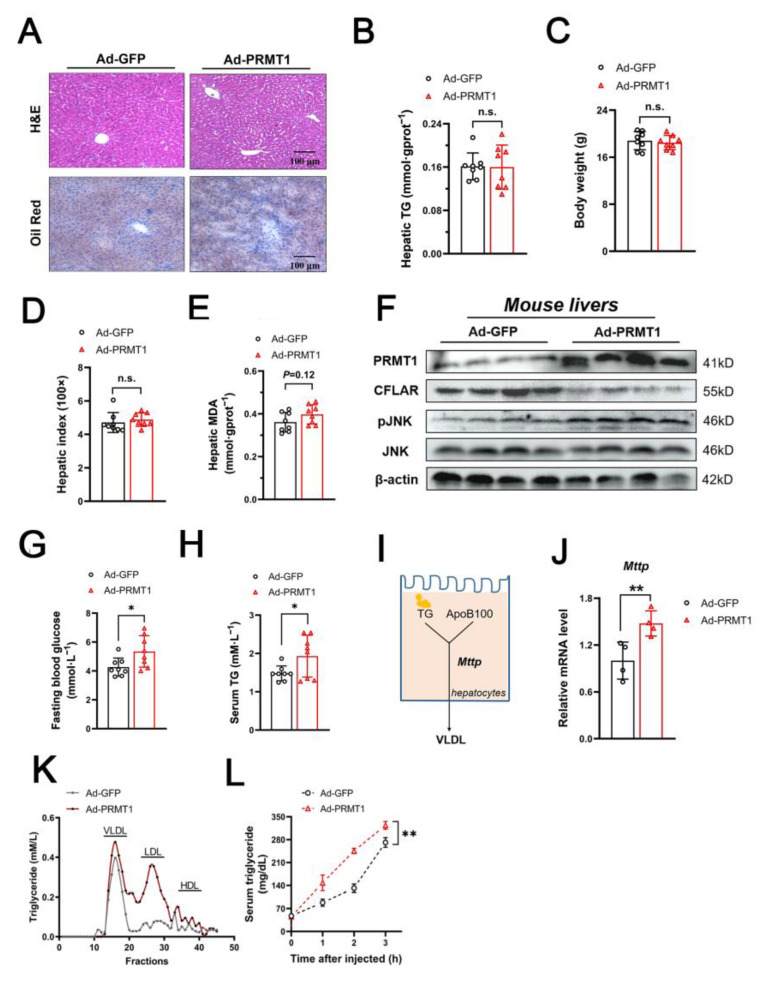
Overexpression of PRMT1 affects systemic lipid homeostasis in C57BL/6J mice. (**A**) Representative images of H&E and oil red O staining. Scale bar = 100 μm. (**B**–**E**) Hepatic triglyceride (TG) content (**B**), body weight (**C**), hepatic index (**D**), and hepatic MDA levels (**E**) were tested (*n* = 8/group). (**F**) Western blot analysis of protein expression of PRMT1 and the CFLAR/JNK axis in livers of mice injected with the indicated adenovirus (*n* = 4/group). (**G**–**I**) Fasting blood glucose levels (**G**), serum TG (**H**), and MTTP-mediated VLDL secretion in hepatocytes (**I**). (**J**) Relative mRNA levels of Mttp in the livers of mice treated as in (**F**) (*n* = 4/group). (**K**) FPLC analysis of serum lipoproteins in mice treated with Ad-GFP or Ad-PRMT1 for seven days. (**L**) Hepatic VLDL secretion analysis in mice treated after injection of tyloxapol (500 mg/kg body weight). Serum was collected, and the triglyceride content was measured using the kit described in the Materials and Methods at the indicated time point. All expressions are normalized to β-actin. A two-tailed Student’s test was used. * *p* < 0.05, ** *p* < 0.01, n.s. means no significance. All data are shown as the mean ± SD.

Given the observation of the beneficial effect of restoring CFLAR expression by interfering with liver PRMT1 in the MCD-fed mouse model, we also purposefully confirmed the significance of this mechanism under normal physiological conditions. To test this idea, adenovirus-expressing PRMT1 was injected into C57BL/6J mice via the tail vein. Unexpectedly, the results from the H&E and oil red O staining indicated no obvious histological abnormalities or lipid accumulation after overexpression of PRMT1 (Figure 8A). Consistently, forced expression of PRMT1 did not affect triglyceride content in the liver, body weight, or the hepatic index (Figure 8B–D). No obvious changes in oxidative stress were observed after the injection of adenovirus-expressing PRMT1, as evidenced by unimpaired MDA content in the liver (Figure 8E). However, overexpression of PRMT1 still decreased the expression of CFLAR but increased the expression of p-JNK (Figure 8F). Interestingly, the Ad-PRMT1-treated mice showed significantly increased serum total triglyceride levels, as well as fasting blood glucose levels, as compared with the Ad-GFP-treated mice (Figure 8G,H). We further investigated the expression of genes involved in lipoprotein assembly and secretion. The results suggested that ectopic expression of PRMT1 promoted the mRNA expression of microsomal triglyceride transport protein (MTTP), a key enzyme in the assembly of VLDL (Figure 8I,J), while it inhibited the mRNA expression of *Ppar-α* (Figure S1A). No changes in the expression of *Srebp-1c* were observed (Figure S1B). Next, the serum lipoprotein composition was analyzed by an FPLC analysis to explain these seemingly contradictory findings. The results showed that forced expression of PRMT1 not only significantly increased serum triglyceride content in VLDL and LDL particles (Figure 8K) but also led to increased liver lipoprotein production and secretion after the injection of Tyloxapol (Figure 8L).

## 3. Discussion

Although there is compelling evidence indicating the involvement of PRMT1 in a variety of liver diseases, the precise physiological role of PRMT1 in hepatocytes remains not fully comprehended [27,28,29,30]. A recent study further revealed that the depletion of *Prmt1* in adipocytes impairs glucose homeostasis in cases of diet-induced obesity [31]. The mRNA and protein expression of PRMT1 exhibited significant upregulation in the kidneys of diabetic rats as well as in patients with type 2 diabetes [32]. Furthermore, it is noteworthy that PRMT1 expression was conspicuously induced in the livers of patients afflicted with NAFLD [21]. Additionally, rats fed an MCD diet for eight weeks showed a marked increase in the protein expression of PRMT1 in the liver, which aligns with our results in Figure 1C [33], while no further work was performed in investigating the potential effects of PRMT1 on the regulation of hepatic lipid metabolism. Our data clearly showed that adenovirus-mediated overexpression of PRMT1 significantly facilitated lipid synthesis and accumulation in both HepG2 and primary hepatocytes. In addition, alcohol intake resulted in significant upregulation of PRMT1 in the livers of mice [27], while PRMT1 knockout reduced the expression of hepatic HNF4α and had a regulatory effect on hepatocyte proliferation. However, there is a need for further evidence to validate the studies above, as they did not investigate whether PRMT1 could potentially regulate lipid or glucose metabolism in the liver [34]. Interestingly, Qiao et al. demonstrated that PRMT1 variant 2 could directly interact with PGC-1α in primary hepatocytes and adipocytes, suggesting that PRMT1 might affect glucose homeostasis and adaptive thermogenesis [22]. Our data clearly demonstrated that overexpression of PRMT1 not only enhanced lipid synthesis and accumulation in HepG2 and primary hepatocytes but also upregulated the expression of gluconeogenic genes including *G6pc*, *Pepck*, and *Fbp1*, subsequently leading to increased fasting blood glucose levels in mice, which was in line with data presented above [22]. In reality, we were unsuccessful in replicating an experimental animal model that accurately demonstrates the complete range of pathophysiological, histological, molecular, and clinical characteristics associated with the progression of NAFLD/NASH in humans. NAFLD represents a complex pathological condition affecting numerous organs and systems throughout the body, often presenting with comorbidities such as obesity, IR (insulin resistance), and hyperlipidemia [35]. When employing a classic model using HFD-fed male C57BL6 mice to mimic NAFLD in vitro, several months are typically required to induce mild liver fibrosis, a telltale sign of NASH progression, while the liver exhibits gradual recovery upon cessation of the high-fat diet [36]. Conversely, the 4–6-week MCD diet promotes significant weight loss and reduces blood glucose levels as well as IR; it also gives rise to pronounced pathological signs of liver fibrosis, which can be challenging to reverse even after discontinuing the diet [37]. Additional studies are needed that involve so-called “non-classic” NAFLD patients, such as those with obvious liver fibrosis but no IR [38] or lean NAFLD [39,40]. Indeed, sufficient basic research is imperative to delve into the metabolic mechanisms associated with various pathological manifestations, thereby comprehensively encapsulating the intricacies of human diseases.

Zhang et al. provided some results suggesting that CFLAR may be involved in CDKN2A-induced lipid accumulation in the NCTC-1469 cell line, lacking methylation test and knockdown experiments under pathological models [41]. Surprisingly, Xu L et al. reported that silencing of hepatic PRMT1 promoted steatosis in the livers of HFD-induced C57BL/6N mice via reduced PGC1α with no obvious changes in serum lipid contents, while overexpression of wild-type PRMT1 alleviated HFD-induced hepatic steatosis [24]. Two reports seemed to produce contradictory conclusions in NAFLD models in vitro and in vivo. However, our results clearly demonstrated that silencing of PRMT1 not only significantly alleviated the disorder of lipid metabolism in mice under the MCD model but also markedly decreased triglyceride content in PA/OA-treated primary hepatocytes. Furthermore, our results showed that overexpression of PRMT1 in the mice livers under normal physiological conditions promoted hepatic VLDL secretion and increased serum triglyceride levels, which implied an increased metabolic burden on the liver. CFLAR was initially discovered to inhibit the cleavage activation of pro-caspase 8, a classic cell apoptosis signaling molecule, representing one of the results of hepatocytes being attacked by a large amount of lipids. For one thing, different cellular processes may serve as indicators of varying disease progression stages; for another, mice with different genetic backgrounds and different variants of PRMT1 may be employed to elucidate and clarify these seemingly contradictory results.

CFLAR is a key suppressor of non-alcoholic steatohepatitis in mice and nonhuman primates [7,8,42]. However, how the hepatocytic CFLAR protein, the main form of functionality so far known, was regulated under physiological and pathological conditions still remains unknown. We presented basic and convincing evidence in the present work to propose that PRMT1 is the upstream regulator of CFLAR in hepatocytes, which promotes its ubiquitination degradation through direct methylation of CFLAR, thus inhibiting the CFLAR-JNK axis and ultimately promoting the occurrence and development of NAFLD. 

Of course, there are some potential and unavoidable limitations to the study, which include the following: (1) Since at least seven splice variants of PRMT1 are identified and these variations can impact enzymatic activity and substrate specificity, it is difficult to achieve isoform-specific inhibition or knockdown of PRMT1 [43]. (2) Each in vivo model used has its specificity and bias, making it difficult to choose a model that fully simulates all the pathological manifestations of NAFLD/NASH in humans. It is necessary to conduct additional experiments under multiple conditions. (3) The precise protein methylation site/sites of CFLAR remains/remain to be further revealed for specific drug recognition. 

Taken together, we demonstrated that hepatic expression of PRMT1 was regulated by nutritional status and dysregulated in diet-induced mouse models of NAFLD. PRMT1 was a lipogenic gene that could promote triglyceride synthesis and accumulation in hepatocytes via directly methylating CFLAR and enhancing its ubiquitination-mediated degradation. In the liver pathological model constructed with the MCD diet, interfering with PRMT1 could effectively alleviate mice hepatic lipid accumulation and inflammatory pathological manifestations. Forcing expression of PRMT1 in the liver increased VLDL secretion and fasting blood glucose levels in normal mice under physiological conditions, suggesting that PRMT1 was a potential therapeutic target of NAFLD.

## 4. Materials and Methods

### 4.1. Animals and Treatment

Male C57BL/6J mice aged six to eight weeks were purchased from the Hubei Provincial Center for Disease Control and Prevention. All animal experiments were approved by the Ethics Committee of Hubei University (approved number: 20220015). The mice were kept in a controlled environment (22 °C, 60% relative humidity, and 12/12 h day/dark cycle). Water and food were provided ad libitum. After a week of adaptive feeding, the mice were randomly divided into two groups, with 8 mice in each group, and injected with purified recombinant adenovirus Ad-GFP/Ad-PRMT1 via the tail vein. Seven days after injection, the mice were sacrificed, and the serum and livers were collected for further analysis. For the knockdown of PRMT1 experiment in vivo under the six-week methionine–choline-sufficient (MCS)/methionine–choline-deficient (MCD) dietary model, the mice were randomly divided into three groups, with 12 mice in each group, and injected with purified adenovirus twice on weeks two and four. At week 6 (42 days), testing was conducted after an overnight fast prior to the termination of the animal experiment. The liver tissue was preserved in liquid nitrogen and fully lysed using RIPA or trizol reagent. For the feeding/fasting/refeeding challenge, the mice were fasted for 48 h or refed a normal diet for another 12 h [44]. Because of the very low levels of serum glucose and insulin, while a large amount of triglycerides derived from peripheral tissue breakdown (instead of short-term 24 h glycogen breakdown) accumulated in the liver, we performed a long-term fasted-challenge, which was similar to the pathological changes induced by the MCD dietary conditions in mice [45,46]. The MCD (lacking L-methionine and choline bitartrate)/MCS diets comprised 16% protein, 62% carbohydrate, and 21% fat per 100 kcal of calories, purchased from Jiangsu Xietong Pharmaceutical Bio-engineering Co., Ltd., Nanjing, China (XTMCD/XTMCS).

Mouse blood was collected, left to stand at room temperature for 2 h, and then centrifuged at 3000 rpm for 15 min at 4 °C. The serum was collected at the supernatant. The AST and ALT levels in the serum were measured according to the instructions (C010-2-1/C009-2-1, Nanjing Jiancheng Bioengineering Institute, Nanjing, China). The total asymmetric dimethylarginine levels in the serum were measured by an ELISA kit (K7828, Immunodiagnostik, Bensheim, Germany) following the instructions [47].

### 4.2. Construction of Recombinant Adenovirus

Ad-GFP, Ad-PRMT1, and Ad-shPRMT1 were constructed using the Ad-Easy system [48]. Briefly, the coding sequences of PRMT1 and the short-hairpin structure targeted PRMT1 were cloned into the pAd-Track-CMV/U6 vector (16405, Addgene, MA, USA). Recombination of the Track and Easy vector was carried out in BJ5183 bacteria. Then, the vector was transfected to 293A cells to obtain recombinant adenovirus. After extensive amplification, cesium chloride density gradient centrifugation was performed to purify adenovirus. Titer determination was carried out according to the manufacturer’s instructions. The mice were injected with the corresponding recombinant adenovirus through the tail vein [48].

### 4.3. Cell Culture and Transfection

The HepG2 cell lines used in the experiments (CL-0103, Procell, Wuhan, China) were tested to be free of mycoplasma contamination and cultured in DMEM medium (Gibco, Billings, MT, USA) supplemented with 10% fetal bovine serum (FBS) and 1% penicillin–streptomycin. The cells were seeded in a well of six-well plates at a quantity of 2–4 × 10^5^ cells. Then, the cells were transfected with PRMT1-Myc (Myc-tag) plasmid using lipofectamine 2000 (11668019, Invitrogen, Carlsbad, CA, USA) and Opti-Mem (31985070, Gibco) according to the instructions the next day for 24 h. A mix of 0.25 mM oleic acid (OA) and 0.5 mM palmitic acid (PA) was added to the medium for another 24 h to establish the in vitro model of lipid accumulation. OA stock solution was prepared and stored in methanol at −20 °C. PA stock solution was stored in ultrapure water at 4 °C and was heated to 60 °C to promote its dissolution. Fatty acid-free bovine serum albumin (BSA, BS260, Biosharp, Hefei, China) was added to the medium at a final concentration of 10% individually as the control group or accompanied with 0.25 mM OA + 0.5 mM PA (PO) as the PO group. Before use, different FFA stock solutions were dissolved in DMEM or RPMI-1640 complete media to yield concentrations as indicated. The culture medium of both groups contained the same 10% FBS. The culture medium was mixed well and placed in the incubator for cultivation.

### 4.4. Isolation, Culture, and Transfection of Primary Hepatocytes

Male C57BL/6 aged six to eight weeks were used for hepatocyte isolation. The mice were fixed after anesthetization. The livers were perfused and digested with Krebs Ringer medium and collagenase. Then, the livers were excised, minced, and filtered through a 70 μm filter. The precipitate cells were washed twice with cold PRMI 1640 medium (Gibco) and seeded into indicated plates [49]. After 6 h, the hepatocytes were transfected with Ad-GFP or Ad-PRMT1 for the following experiments. Because of the difficulty in the proliferation of primary hepatocytes in vitro, the hepatocytes were infected with a low-degree adenovirus stock solution of about 5–10 microliters. The infection was verified by observing the expression of green fluorescent protein (GFP) through a fluorescence microscope after 24 h.

### 4.5. Western Blot Analysis

Total protein was isolated from cells or tissue using RIPA lysis buffer (containing 0.1% SDS) with protease (4693132001, Roche, Basel, Switzerland) and phosphatase inhibitor (4906837001, Roche). Protein was quantified using a BCA kit (23227, Thermo, Waltham, MA, USA). Total protein was divided into 20 μg for cells or 50 μg for tissues and denatured with SDS-PAGE loading buffer at 95 °C for 10 mins. Then, the protein samples were separated by 8–12% SDS-PAGE and transferred to PVDF membranes. After blocking with 5% skim milk diluted in TBST, the membranes were washed twice and incubated with primary antibodies (1:1000) at 4 °C overnight. The manufacturer of the antibodies can be found in Supplementary Table S1. The next day, the membranes were incubated with the corresponding secondary antibodies at a dilution ratio of 1:10000 (A21010 and A21020, Abbkine, Atlanta, GA, USA) at room temperature for 1 h. Lastly, covered with ECL reagent, the membranes were detected for grayscale scanning and quantification using Image J (version 1.8.0). 

### 4.6. Immunoprecipitation (IP) Assay

Cells were transfected with indicated plasmid or adenovirus for 24 h and lysed in ice-cold IP RIPA buffer (lacking SDS) with protease and phosphatase inhibitor for 30 min. The total protein solution was removed by centrifugation at 4 °C. For each IP assay, a 200–300 μL sample was incubated with 20–30 μL Protein A/G beads (sc-2003, Santa cruz, CA, USA) and 1–1.5 μg primary antibody at 4 °C overnight gently. The next day, the IP medium was washed 4–5 times with cold IP buffer, with 2 × SDS-PAGE loading buffer added the last time. All media were heated at 95 °C for 10 min. The supernatant was used for further Western blot analysis. 

### 4.7. Ubiquitination Assays 

Protease and phosphatase inhibitors were included in the lysis. Additionally, MG132 (HY-13259, MCE, Monmouth Junction, NJ, USA) was added to the cell medium before being harvested. Mice primary hepatocytes were lysed with IP lysis buffer containing 1% SDS and were then denatured by heating at 95 °C for 10 min. The supernatants were then diluted with IP lysis buffer to an SDS concentration of 0.1%. After the lysates were centrifuged, the supernatants were subjected to the IP assays described above. The next day, the beads–protein complex was boiled in 2× SDS-PAGE loading buffer and tested by Western blotting.

### 4.8. Quantitative Real-Time PCR

Total RNA was extracted using TRIzol reagent (15596026CN, Invitrogen) according to the manufacturer’s instructions. Then, cDNA was synthesized with 4 μg RNA using an RNA reverse transcription kit (FSQ-101, Toyobo, Osaka, Japan). The BIO-RAD CFX96 instrument and attached software tools were used to quantify the amplification products with SYBR Green fluorescent dye (QPK-201, Toyobo). Gene expression was normalized to that of a housekeeping gene (ACTB) and calculated using the comparative ΔΔCT method. The related primers are listed in Supplementary Table S2.

### 4.9. FPLC Analysis of Serum Lipoprotein Distribution

Plasma from three mice per group was pooled together and subjected to fast protein liquid chromatography (FPLC) analysis [50]. Serum lipoproteins were separated at a rate of 0.3 mL/min with a Superose-6 Increase 10/300 GL column and 0.3 mL for each fraction. Then, the fractions were collected for the analysis of triglyceride content by assay kits (A110-1-1, Nanjing Jiancheng Bioengineering Institute).

### 4.10. Hepatic VLDL Secretion Assay

The serum very low-density lipoprotein (VLDL) production was measured in overnight-fasted mice injected with tyloxapol (500 mg/kg body weight, MCE). Serum samples were collected by tail bleeding at indicated time points for triglyceride measurement. 

### 4.11. Statistical Analysis

All data are from at least three independent repeated experiments. Statistical analyses were performed using GraphPad Prism v8. Variances were assessed with the Bartlett test for normally distributed data. For the comparison of multiple groups with normal distribution and equal variance, data in groups were compared using a two-tailed unpaired Student’s *t* test in two groups or one-way ANOVA in more groups. Values of *p* < 0.05 were considered statistically significant.

## Data Availability

Data will be made available on request.

## Supplementary Materials

The following supporting information can be downloaded at: https://www.mdpi.com/article/10.3390/ijms25147897/s1.

## Abbreviations

NAFLD	non-alcoholic fatty liver disease
NASH	non-alcoholic steatohepatitis
PRMT1	protein arginine methyltransferase 1
CFLAR	caspase 8 and fadd-like apoptosis regulator
JNK	c-Jun N-terminal kinase
CCL2	chemokine (C-C motif) ligand 2
SREBP-1C	Sterol regulatory element binding proteins-1c
PNPLA3	patatin-like phospholipase domain containing 3
FAS	fatty acid synthase
ACOX1	acyl-coenzyme A oxidase 1
PPAR-α	peroxisome proliferator-activated receptor
CPT1α	carnitine palmitoyltransferase 1
MCAD	medium chain aryl-CoA dehydrogenase
UCP2	uncoupling protein 2
COL1A1	collagen type I alpha 1 chain
IL-1β	interleukin 1 beta
PDK4	pyruvate dehydrogenase kinase 4
PO	OA (oleic acid) + PA (palmitic acid)
TG	triglyceride
TC	total cholesterol
MTTP	microsomal triglyceride transfer protein
TNF-α	tumor necrosis factor-α
PEPCK	phosphoenolpyruvate carboxykinase
G6PC	glucose-6-phosphatase
FBP1	fructose-1,6-bisphosphatase 1
IL-6	interleukin 6
MMA	monomethylation
ADMA	asymmetric dimethylarginine
FPLC	fast protein liquid chromatography
PGC-1α	peroxisome proliferative activated receptor gamma coactivator 1α
ACC1	acetyl-CoA carboxylase 1
CD36	cluster of differentiation 36
GPAT	glycerol-3-phosphate acyltransferases
