# Supplementary material for: The Regulatory Impact of CFLAR Methylation Modification on Liver Lipid Metabolism
Source: Int J Mol Sci. 2024 Jul 19;25(14):7897. doi: 10.3390/ijms25147897 (PMC11277202; doi:10.3390/ijms25147897)
Supplement: Supplementary file 1 [file ijms-25-07897-s001.zip › ijms-3079769-supplementary.pdf]

The regulatory impact of CFLAR methylation modification on liver lipid metabolism

Chen Ye, Wen Jiang, Ting Hu, Jichao Liang,*Yong Chen *

National & Local Joint Engineering Research Center of High throughput Drug Screening Technology, Hubei Province Key Laboratory of Biotechnology of Chinese Traditional Medicine, Hubei University, Wuhan 430062, China.

Author. E-mail: 202311107010121@stu.hubu.edu.cn (C, Y)

*Corresponding author. E-mail: liang529114@163.com (J. L) or 20120083@hubu.edu.cn ; cy101610@hubu.edu.cn (Y.C).

Supplementary tables:

Table S1. Antibody reagents information

Anti-targets	Source	Cat.
CFLAR	Cell signaling technology, U.S.A.	#56343
PRMT1	Cell signaling technology, U.S.A.	#2449
JNK	Cell signaling technology, U.S.A.	#9252
pJNK	Wanleibio, Shenyang, China	WL01813
ubiquitin	Cell signaling technology, U.S.A.	#3933
ADMA	Cell signaling technology, U.S.A.	#13522
β-actin	Santa cruz, U.S.A.	sc-47778

Table S2. Primers for quantitative PCR

Primers for mouse	Sequences（5’-3’）
β-actin	F: AACCGTGAAAAGATGACCCAGAT
	R: CACAGCCTGGATGGCTACGTA
Prmt1	F: AACATGCAGAGGATGCCAGT
	R: ACTCCATGTTTCACAATCGGCA
Cflar	F: CTGTGTCTGCCGAGGTCATTC
	R: AGAGCAATTCAGCCAAGGTAGC
Ppar-α	F: TATTCGGCTGAAGCTGGTGTAC
	R: CTGGCATTTGTTCCGGTTCT
Cpt-1α	F: AGGACCCTGAGGCATCTATT
	R: ATGACCTCCTGGCATTCTCC
Acox1	F: GTCTCCGTCATGAATCCCGA
	R: TGCGATGCCAAATTCCCTCA
Mcad	F: AAACATGGGCCAGCGATGCTCT
	R: AGGGCATACTTCGTGGCTTCGT
Pdk4	F: TTCACACCTTCACCACATGC
	R: AAAGGGCGGTTTTCTTGATG
Srebp-1c	F: CACTTCTGGAGACATCGCAAAC
	R: ATGGTAGACAACAGCCGCATC
Fas	F: CTGCGGAAACTTCAGGAAATG
	R: GGTTCGGAATGCTATCCAGG
Acc1	F: GGCCAGTGCTATGCTGAGAT
	R: AGGGTCAAGTGCTGCTCCA
Pnpla3	F: ATTCCCCTCTTCTCTGGCCTA
	R: ATGTCATGCTCACCGTAGAAAGG
Gpat	F: CCATTGTGGAGGATGAAGTG

	R: TGGATCGTGCCAGATAGGGA
Cd36	F: TGGGTTTTGCACATCAAAGA
	R: GATGGACCTGCAAATGTCAGA
Mttp	F: GCTAAGAAGCTGATAATGGGAGG
	R: CCACTCTTGGAGAAACGGTCATA
G6pc	F: TCTGTCCCGGATCTACCTTG
	R: GCTGGCAAAGGGTGTAGTGT
Pepck	F: TGCCCCAGGCAGTGAGGAAGTT
	R: GTCAGTGAGAGCCAGCCAACAGT
Fbp1	F: GCATCGCACAGCTCTATGGT
	R: ACAGGTAGCGTAGGACGACT
Ccl2	F: TACAAGAGGATCACCAGCAGC
	R: ACCTTAGGGCAGATGCAGTT
Tnf-α	F: CATCTTCTCAAAATTCGAGTGACAA
	R: TGGGAGTAGACAAGGTACAACCC
Il-1β	F: GGGAACGTCACACACCAGCA
	R: CCGTGGACCTTCCAGGATGA

Primers for homo	Sequences（5’-3’）
β-actin	F: TCACCCACACTGTGCCCATCTACGA
	R: CAGCGGAACCGCTCATTGCCAATGG
Prmt1	F: TGCGGTGAAGATCGTCAAAGCC
	R: GGACTCGTAGAAGAGGCAGTAG
Cflar	F: GGACTTGGCTGAACTGCTCTAC
	R: TCCAAATCCTCACCAATCTCTG
Ppar-α	F: CTGGAAGCTTTGGCTTTACG
	R: ACCAGCTTGAGTCGAATCGT
Cpt-1α	F: TCCAGTTGGCTTATCGTGGTG
	R: TCCAGAGTCCGATTGATTTTTGC
Acox1	F: GACCTGAGTGAACTGCCTGAG
	R: CACCACAAGCCATCCGACAT
Gpat	F: CCATTGTGGAGGATGAAGTG
	R: TGGATCGTGCCAGATAGGGA
Tnf-α	F: CCTCTCTCTAATCAGCCCTCTG
	R: GAGGACCTGGGAGTAGATGAG
Il-6	F: CCAGGAGCCCAGCTATGAAC
	R: CCCAGGGAGAAGGCAACTG
Il-1β	F: TCCAGGGACAGGATATGGAG
	R: TCTTTCAACACGCAGGACAG
Ccl2	F: GCTCAGCCAGATGCAATCAA
	R: TTCTTTGGGACACTTGCTGC

Supplementary figures:

Figure S1

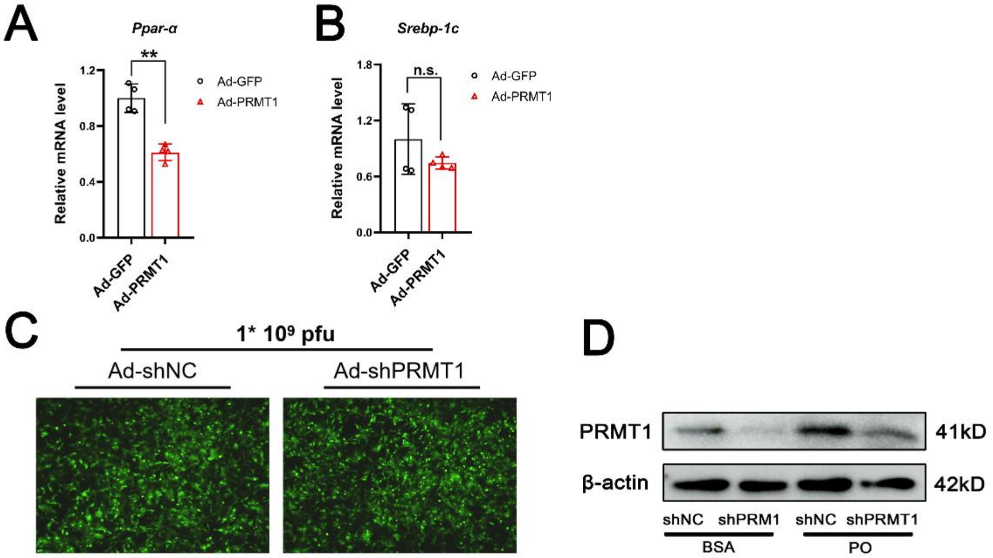

Fig. S1 The effect of Ad-PRMT1 on liver mRNA and the knockdown efficiency of Ad-shPRMT1 on primary hepatocytes. (A, B) Relative mRNA expression of Ppar-α (A) and Srebp-1c (B) in the mice liver (n=4) infected with Ad-GFP or Ad-PRMT1. (C) The fluorescence images of primary hepatocytes infected with Ad-shNC or Ad-shPRMT1 (1×10^9 pfu) for 24 h. (D) Western blot analysis of protein expression of PRMT1 in primary hepatocytes infected with Ad-shNC or Ad-shPRMT1 in the presence/absence of PO. Two-tailed student's t test was used. *P<0.05, **P<0.01, n.s., not significant. Data were shown as the mean ± SD.

Figure S2

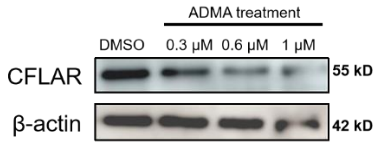

Fig. S2 The effect of ADMA treatment on endogenous CFLAR protein levels in HepG2 cells. HepG2 cells were treated with DMSO or Various concentrations of ADMA(0.3μM, 0.6μM and 1μM). After 24 h, cells were lysed and total protein were used to western blot analysis.
